# Pathogen Populations Evolve to Greater Race Complexity in Agricultural Systems – Evidence from Analysis of *Rhynchosporium secalis* Virulence Data

**DOI:** 10.1371/journal.pone.0038611

**Published:** 2012-06-18

**Authors:** Jiasui Zhan, Lina Yang, Wen Zhu, Liping Shang, Adrian C. Newton

**Affiliations:** 1 Key Lab for Biopesticide and Chemical Biology, Ministry of Education, Fujian Agriculture and Forestry University, Fuzhou, China; 2 Fujian Key Lab of Plant Virology, Institute of Plant Virology, Fujian Agriculture and Forestry University, Fuzhou, China; 3 The James Hutton Institute, Invergowrie, Dundee, Scotland, United Kingdom; University of the West of England, United Kingdom

## Abstract

Fitness cost associated with pathogens carrying unnecessary virulence alleles is the fundamental assumption for preventing the emergence of complex races in plant pathogen populations but this hypothesis has rarely been tested empirically on a temporal and spatial scale which is sufficient to distinguish evolutionary signals from experimental error. We analyzed virulence characteristics of ∼1000 isolates of the barley pathogen *Rhynchosporium secalis* collected from different parts of the United Kingdom between 1984 and 2005. We found a gradual increase in race complexity over time with a significant correlation between sampling date and race complexity of the pathogen (r_20_ = 0.71, p = 0.0002) and an average loss of 0.1 avirulence alleles (corresponding to an average gain of 0.1 virulence alleles) each year. We also found a positive and significant correlation between barley cultivar diversity and *R. secalis* virulence variation. The conditions assumed to favour complex races were not present in the United Kingdom and we hypothesize that the increase in race complexity is attributable to the combination of natural selection and genetic drift. Host resistance selects for corresponding virulence alleles to fixation or dominant frequency. Because of the weak fitness penalty of carrying the unnecessary virulence alleles, genetic drift associated with other evolutionary forces such as hitch-hiking maintains the frequency of the dominant virulence alleles even after the corresponding resistance factors cease to be used.

## Introduction

The evolution of race complexity, here defined as the number of differential cultivars a pathogen isolate infects, has been one of main concerns of plant pathologists, plant breeders and farmers in devising strategies of resistance gene deployment. Complex races, as compared to simple races, have the ability to infect more cultivars with single resistance gene deployed in monocultures or mixtures, or cultivars with multiple resistance genes, leading to simultaneous loss of many host resistance genes [1], [2], [3]. The emergence of complex races in pathogen populations is therefore thought unlikely to occur unless selective regimes favouring the complex races are present recurrently and the benefits from this positive host selection are large enough to compensate for the cost of carrying unnecessary virulence alleles. Our understanding of evolution in race complexity is generally inferred from theoretical work or short-term and small scale field observations. Experimental test and verification of evolutionary theory including the evolution of race complexity through long-term observation of population dynamics under large farm field conditions is limited.

Due to important impacts of pathogens on the commercial life of resistant cultivars and the requirement for increased use of pesticides when resistance in commercial cultivars ceases to be effective, several countries established virulence surveys in 1960s and 1970s to monitor temporal race dynamics of some major crop pathogens. The United Kingdom Cereal Pathogen Virulence Survey (UKCPVS) (www.hgca.com) has sustained continuity for most pathogens including *Rhynchosporium secalis* until 2005. Analysis of pathogen data from these surveys can not only provide plant breeders/pathologists with information and material for resistance screening, but also can be very useful for studying the evolution of plant pathogens because many of these surveys cover large geographic areas and continue for several decades [4], [5], [6].

Barley leaf blotch or scald is one of the most destructive diseases of barley crops. The disease occurs in all of major barley growing regions in the world and can cause significant reductions in barley yield and malting quality [7]. Population genetic studies using molecular and morphological markers reveal that *R. secalis*, the causal agent of leaf blotch, is a highly variable pathogen [8], [9], [10], possibly attributing to its large effective population size [11], high gene flow [8], [12], high mutation rate [13], [14], frequently sexual reproduction [9] and somatic recombination [15], [16]. These studies also show little genetic differentiation among *R. secalis* populations collected from the same countries or regions [8], [9], [17], [18]. Recently, the pathogen was re-named as *R. commune* [19].

Barley resistance to *R. secalis* can be race-specific or race-non-specific [20]. Both types of resistance affect pre- and post-penetration stages of pathogen development but in different ways. Race-specific interaction follows the ‘gene-for-gene’ model [21]. Incompatible interactions occur between barley major resistance genes [22], [23] and their corresponding *R. secalis* avirulence alleles, resulting in a resistant phenotype. Resistant genotype in this case does not necessarily indicate a lack of pathogen invasion and colonization [20]. *Rrs1*-mediated resistance, for example, allows some infection even in resistant interactions but symptom expression does not occur [24]. In compatible interactions, virulence or avirulence allele products interact with specific host targets, resulting in a susceptible phenotype. Both race-specific and race-non-specific resistance to *R. secalis* have been identified and incorporated into commercial cultivars to control the disease (see [20] for review], but high genetic variability in *R. secalis* populations can result in rapid adaptation of the pathogen to render resistant cultivars ineffective [7], [25], [26].

A standard set of seven barley differentials were used in the UKCPVS *R. secalis* survey since 1984. From 1984 to 2005, a total of 945 *R. secalis* isolates randomly collected from various parts of the UK were included in the survey [27]. The objectives of this study are to investigate the evolutionary trends of race complexity in *R. secalis* populations and to infer their underpinning genetic mechanisms by reanalyzing the data.

## Materials and Methods

Barley leaves infected with *R. secalis* were obtained from agricultural researchers and advisors around the UK from 1984 to 2005 [27]. Procedures of fungal isolation and virulence characterizations were described in the survey reports [27] and can be found in related publications [28]. The recent survey reports are available at Home Grown Cereals Authority (HGCA) website (www.hgca.com). The survey reports more than 10 years old can be accessed through public libraries such as the British Library and institutional libraries such as the James Hutton Institute library and the Rothamsted Research library. Briefly, infected barley leaves were obtained primarily from agricultural researchers, growers and advisors around UK. Pathogen strains isolated from these infected leaves were used to inoculate the standard seven differentials (i.e. indicator cultivars that can distinguish pathogens with different virulence alleles) in glasshouse seedling tests. Each differential expresses a different resistance phenotype to *R. secalis* challenge and is designated as a Barley Rhynchosporium Resistance (BRR) factors. The differentials were defined simply because they showed differential response to isolates [17], [29], [30]. The genetic basis of the resistance phenotype is not known and therefore matching virulence in the pathogen is similarly known as Barley Rhynchosporium Virulence (BRV) factors. The *R. secalis* survey in the UK was reported continuously from 1968 but the standard set of seven differentials was not established until 1984. In 1997, an eighth differential was added to the standard set. Because this differential was only used for last nine years, it was not included in data analysis. Elwyn R. L. Jones (IGER, Wales, UK) carried out all the *R. secalis* isolations and characterizations throughout this period, ensuring comparability and consistency of the data.

Disease severity was evaluated by taking account both of infection percentage and reaction types using a 0 to 4 scale [31]. An isolate was considered to carry virulence towards a specific differential if overall leaf area with symptoms was over 10%. Race of an isolate was assigned using octal nomenclature [32], [33].

Race complexity in an isolate was determined according to the number of differentials on which the isolate formed symptoms. A complexity index of “7” indicates that the isolate expresses symptoms on all seven differentials (Armelle, Astrix, Athene, Igri, La Mesita, Osiris and Pirate) while a complexity index of “0” indicates that the isolate cannot express symptoms on any of the seven differentials. All isolates carrying the same number of virulence alleles were treated as the same complexity regardless of what specific differentials they expressed symptoms on. With seven differentials, there will be eight potential types (0–7) of race complexity. These race complexities were divided into two groups, each containing an equal number of four types. Isolates with the complexity index of three or less (0, 1, 2, and 3) were grouped into “simple race” while isolates with the complexity index of four or higher (4, 5, 6, and 7) were grouped into “complex race”.

Virulence and race frequency were tabulated yearly and their temporal dynamics over the survey period was evaluated by contingency χ^2^ test [34] and Simple Moving Average analysis [35] across four consecutive years. Number and proportions of barley cultivars being grown during the survey period were taken from the Home Grown Cereals Authority (HGCA) database. The potential number of races presenting in *R. secalis* populations but not detected was estimated using the method described by Huang and Weir [36]. Race and host diversities, the probabilities of two races or cultivars drawn randomly from populations being different, were measured with Nei’s genetic index [37] according to the frequency present in each race or proportion taken by each cultivar. The association between sampling points and race complexity was evaluated using simple linear correlation [38]. The effect of barley host on race dynamics in *R. secalis* was evaluated according to: 1) the correlation between the number of races found and the number of cultivars being grown in each year; 2) the correlation between race diversity and cultivar diversity in each year.

## Results

**Figure 1:**
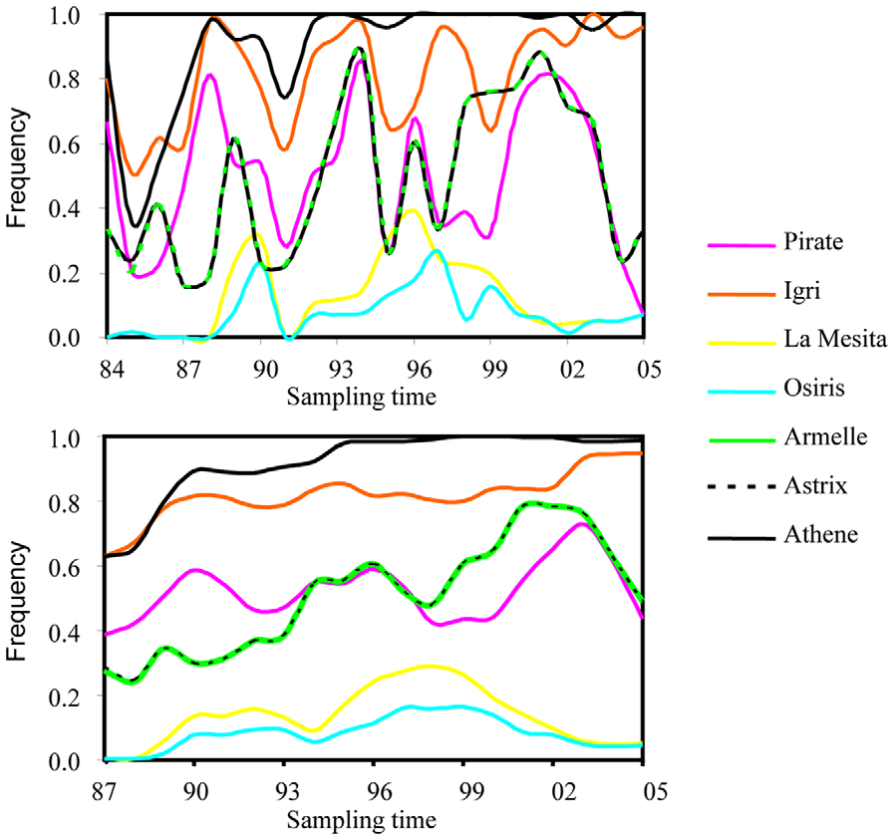
Virulence dynamics of *R. secalis* in United Kingdom between 1984 and 2005. a) The frequency of virulence to the seven differentials; b) Moving average of virulence frequency across four consecutive years.

Frequencies of virulence to the seven differentials changed significantly over the survey period (p<0.0001 for all seven virulence alleles). There was a pattern of cycles for all seven virulence alleles except virulence to Athene (Figure 1). Virulence to Athene was at a persistently high frequency and its frequency in the UK became nearly fixed after 1995. Frequencies of virulence to Armelle and Astrix overlapped in the majority of years.

**Figure 2:**
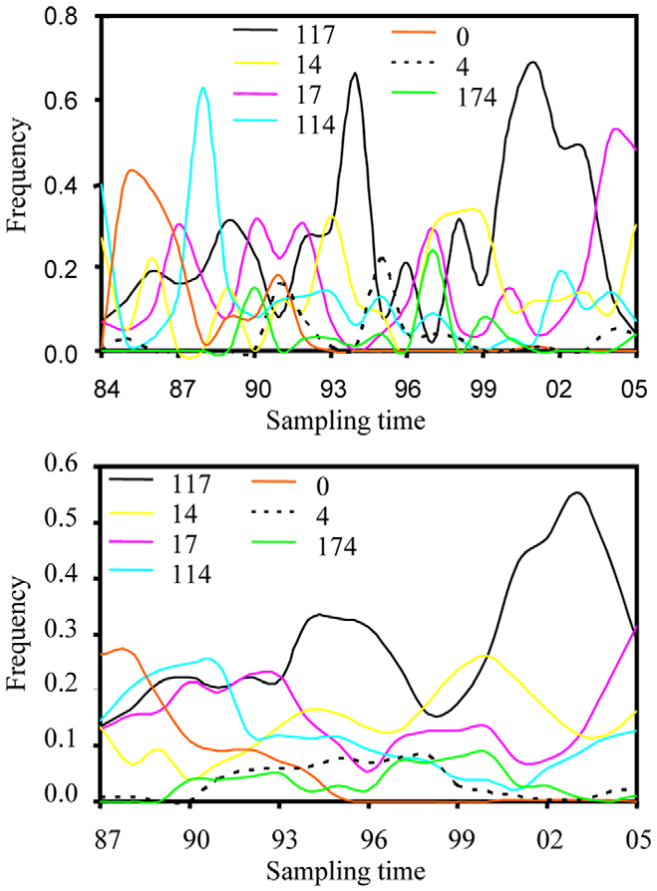
Race dynamics of *R. secalis* in United Kingdom between 1984 and 2005. Only the most common seven races were presented. a) The frequency of races; b) Moving average of race frequency across four consecutive years.

Frequencies of pathogen races changed significantly over the survey period (p<0.0001). Race frequencies displayed periodicity (Figure 2) but with a less clear pattern than virulence alleles (Figure 1). The number of races detected in each year ranged from 4 to 14 with an average of 8.7. The correlation between the number of *R. secalis* isolates assayed each year and the number of races identified in that year was not statistically significant (r_20_ = 0.20, p = 0.3645). A total of 32 races were detected among the 945 isolates over the entire survey. In addition, another 29 races were likely to be present in the population but were not sampled, bringing the total number of races to 61.

**Table 1:** The list of the most common races and their virulence compositions.

Race	Differential Cultivar						
	Pirate	Osiris	La Mesita	Igri	Athene	Astrix	Armelle
0	−*	−	−	−	−	−	−
4	−	−	−	−	−	−	+**
14	−	+	−	−	−	−	+
17	−	+	−	−	+	+	+
114	+	+	−	−	−	−	+
117	+	+	−	−	+	+	+
174	+	+	+	+	−	−	+

* Incompatible reaction, indicating the race does not cause disease to the differential.

** Compatible reaction, indicating the race can cause disease to the differential.

**Figure 3:**
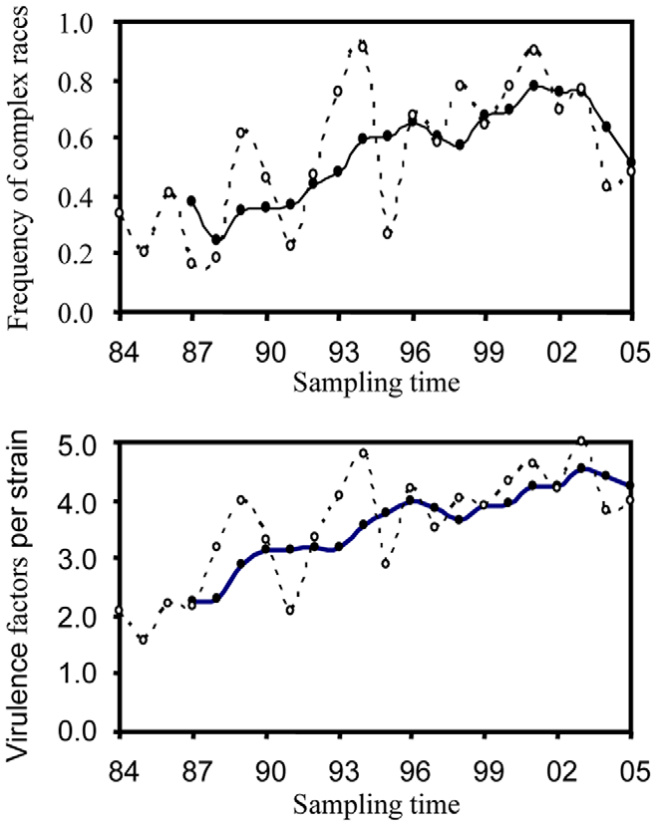
Change of race complexity over time in the UK population of *R. secalis.* The dashed line represents the average number of virulences a pathogen strain carries in each year and the solid line represents a moving average across four consecutive years.

The sign test indicates that simple races dominated eight out of nine surveys conducted between 1984 and 1992 (p = 0.039) while complex races dominated 11 out of 13 surveys conducted between 1993 and 2005 (p = 0.023). Race 0 (no virulence to any differentials, Table 1) dominated the initial three surveys (1985, 1986 and 1987 surveys); race 114 (virulence to three differentials) dominated the 1988 survey and race 117 (virulence to five differentials) dominated the majority of the surveys conducted after 1989 years. The average number of virulence alleles a pathogen strain carries increased over time with a slope of 0.12 and a correlation coefficient of 0.71 (D.F. = 20, p = 0.0002) between sampling point (year) and race complexity of the pathogen (Figure 3).

**Figure 4:**
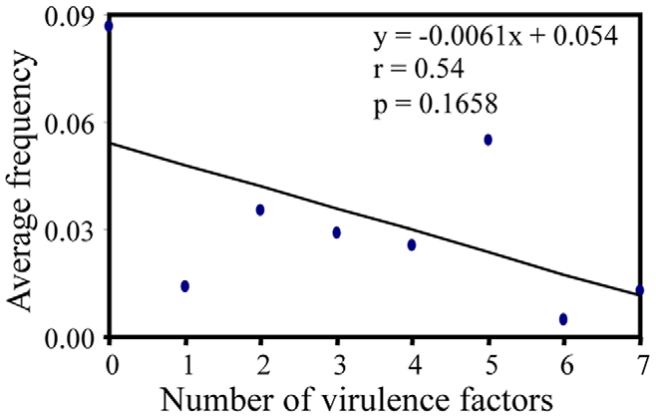
Correlation between race complexity of *R. secalis* isolates and their frequencies in the population.

**Figure 5:**
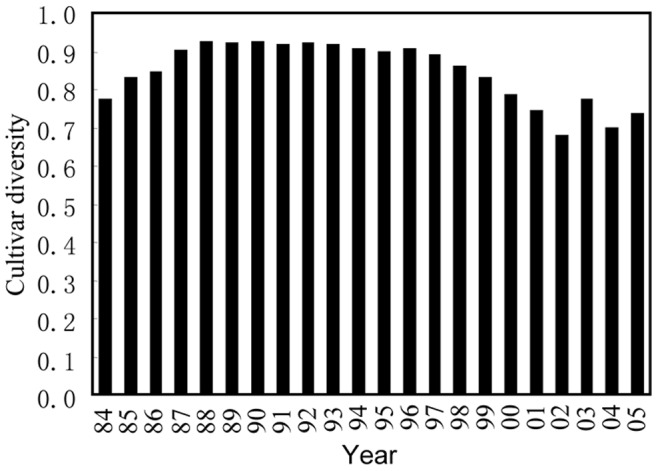
Cultivar diversity in UK barley population between 1984 and 2005.

**Figure 6:**
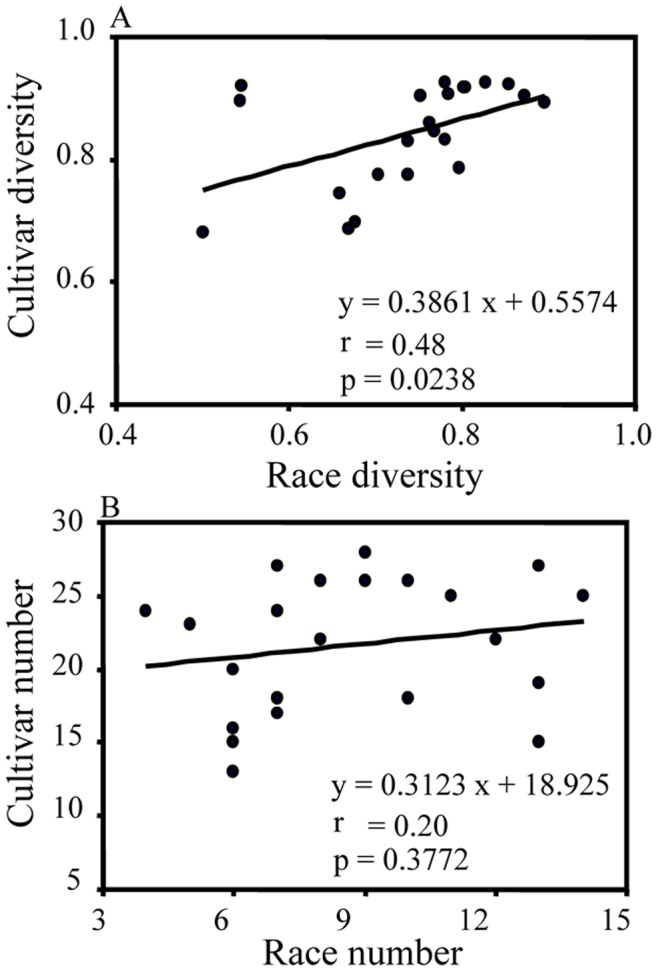
Correlation between host and pathogen variation : A) race and cultivar diversity; B) the number of races detected and the number of cultivars being grown.

The correlation between race complexity and its frequency in the *R. secalis* population was negative but not significant (Figure 4, r_6_ = −0.54, p = 0.1658) when isolates from different years were pooled together to form a single population. When data from different years was analysed individually, an equal opportunity of observing a positive and a negative correlation was found (χ^2^ = 0.02, D.F. = 1, p = 0.8978). Out of the 22 total correlation coefficients, 12 were negative and 10 were positive but none of them were statistically significant.

There was no systematic change in host diversity over the two decades (Figure 5). The correlation between race diversity and cultivar diversity across the survey period was positive and highly significant (Figure 6, r = 0.48, D.F. = 20, p = 0.0238). Though the correlation between number of races found and number of cultivars being grown during this period of time was also positive, it is not statistically significant (Figure 6B, r = 0.20, D.F. = 20, p = 0.3772).

## Discussion

We re-analyzed virulence characteristics of nearly 1000 *R. secalis* isolates published earlier [27] to investigate population genetic dynamics of race structure in plant pathogens in an agricultural system. These isolates were sampled from various parts of the UK over 22 consecutive years and evaluated on a standard set of seven barley differentials. To the best of our knowledge, this represents one of a few attempts to understand the evolution of plant pathogens through analyzing historic virulence data generated from a large geographic area and a long time scale. Our analysis showed the majority of races and virulence alleles in *R. secalis* populations displayed cycles over the survey period (Figure 1 and 2) and we believe the driving force for the cycles is host selection [26], [28], [39], [40], [41]. Few of the resistance genes in the commercial cultivars used during the survey period in the UK were known but data on the number of cultivars and their growing areas in each year were available. When we correlated these host data to the pathogen data, we found there were positive and significant correlations between cultivar diversity and race diversity (Figure 6).

With the seven differentials, there would be 128 (2^7^) potential races in the *R. secalis* populations. However, only about 61 (32 sampled and 29 not sampled) races were present in the populations over the entire survey, accounting for less than a half of the theoretical maximum. There are two likely explanations for this less than expected race richness: 1) although many molecular surveys inferred that sexual recombination could be one of main factors accountable for high genetic variation in *R. secalis* [9], [10], [12], [17], no sexual stage has yet been observed. Lack of random mating constrained the formation of all races; 2) natural selection coupled with genetic drift eliminated less fit races from the population.

The most interesting result we found from the analysis of this historic survey was that *R. secalis* population has gradually increased in race complexity over the survey period (Figure 3). The average number of differentials a pathogen strain can infect increased from 2.1 in 1984 to 4.0 in 2005, gaining a mean of 0.1 differentials each year. This is contradictory to earlier results showing that simple races displayed higher competitive ability than complex races in *R. secalis* and the pathogen populations shifted away from complex race at the end of the experiment [28], [42]. These earlier results were drawn from experiments conducted under controlled greenhouse condition during a single cycle of infection.

Virulence to Armelle and Astrix had the same frequency in the majority of the surveys. It could be argued that the two differentials carry the same resistance gene(s) and including both virulences in data analysis may bias up the estimate of race complexity. However, previous results do not agree with this argument [17], [29], [30], [41]. When the same set of differentials was used to evaluate virulences of *79 R. secalis* sampled from Tunisia, differential Armelle was susceptible to more than 50% of the isolates while differential Astrix was only susceptible to less than 20% of the isolates [30], indicating that the two differentials carry different resistance genes. The pattern of increasing race complexity over time did not change when either Armelle or Astrix was excluded from the analysis (data not shown).

Virulence of *Blumeria graminis*, the causal agent of barley powdery mildew were also characterized in UKCPVS, but only data from 1994 to 2005 were comparable. When we conducted a similar analysis on these data, we also found a gradual increase of race complexity over the 12 years, with a correlation coefficient of 0.84 (D.F = 11, p = 0.0004) between the race complexity and sampling point (year) and an average loss of avirulence alleles (corresponding to an average gain of 0.14 virulence alleles) for each isolate per year. It seems that gradual increase in race complexity over time may not be a unique phenomenon to *R. secalis,* but reflect underlying trends in the evolution of plant pathogenic fungi in agricultural systems. Accumulation of race complexity also was reported in other plant-pathosystems including groundsel-*Erysiphe fischeri* [43], potato-*Phytophthora infestans* [44] and *Linum marginale-Melampsora linit* [45], [46].

The nature of evolutionary mechanisms accounting for this gradual shift from simpler to more complex races in the UK agricultural system is not clear. It has been demonstrated that the presence of conditions (selective regimes) favouring complex races such as extensive use of R gene pyramids, cultivar mixtures, or breeding materials from composite crosses could cause such selection [39]. Engineering several resistance genes into a single cultivar or use of cultivar mixtures has been thought to provide many advantages over traditional approaches of deploying single resistance genes for sustainable disease management [47] but has been adopted rarely by producers on a commercial scale, with the exception of using it to control rice blast in China [48]. In the UK, *R. secalis* has been controlled primarily by cultivars with single resistance genes in the spring crop or with partial resistance in the winter crop, complemented with the application of fungicides [20]. Though the main barley cultivars used over the two decades covered by this survey have changed dramatically, there were no systematic increases in host diversity (Figure 5) and the number of cultivars used during this period of time [49], suggesting lack of selective regimes favouring complex races in this country.

Usually a cultivar with any resistance gene is grown until farmer’s demand stops often at the time when its efficacy for controlling a disease breaks-down. At this stage, the virulence rendering resistance ineffective could have become dominant or even fixed in pathogen populations. Fitness cost of carrying unnecessary genes has been assumed to be the main constraint for the establishment of complex races [50], [51], [52], [53] but may vary substantially across different plant-pathosystems [31], [46], [51], [53], [54], [55]. Consistent with previous results [42], we also found a negative correlation between race complexity and fitness in *R. secalis*-barley, but it seems that this fitness penalty is low in *R. secalis*-barley [56]. With the low fitness cost for carrying unnecessary virulence in *R. secalis* (Figure 4), genetic drift associated with other evolutionary forces such as hitch-hiking selection could maintain dominant virulence alleles at high frequency even when their corresponding resistance genes are withdrawn from commercial use, leading to a gradual build-up of virulence alleles in *R. secalis* populations.

The finding that pathogens evolve from simpler race structure to more complex race structure in agricultural systems has many implications for disease management. Selection for complex races in pathogen populations could cause a great risk for resistance gene deployment strategies such as ‘resistance gene pyramiding’ and cultivar mixtures. Engineering a series of isogenic lines differing only in resistance genes and using them alternatively in time (resistance gene rotation) could be another option. Temporary withdrawal of a resistance gene from commercial use before its corresponding virulence allele becomes dominant might favour negative selection and genetic drift to wipe out the virulence allele, which may not only prevent the accumulation of virulence alleles in pathogen populations but also maximize the life span of the resistance gene as evolution of virulence to this specific resistance gene might have to re-start from the beginning when it is re-used.
